# Supplementary material for: Enhanced production of recombinant HALT-1 pore-forming toxin using two-step chromatographic procedure
Source: MethodsX. 2023 Feb 11;10:102073. doi: 10.1016/j.mex.2023.102073 (PMC9971028; doi:10.1016/j.mex.2023.102073)
Supplement: Supplementary file 1 [file mmc1.pdf]

A

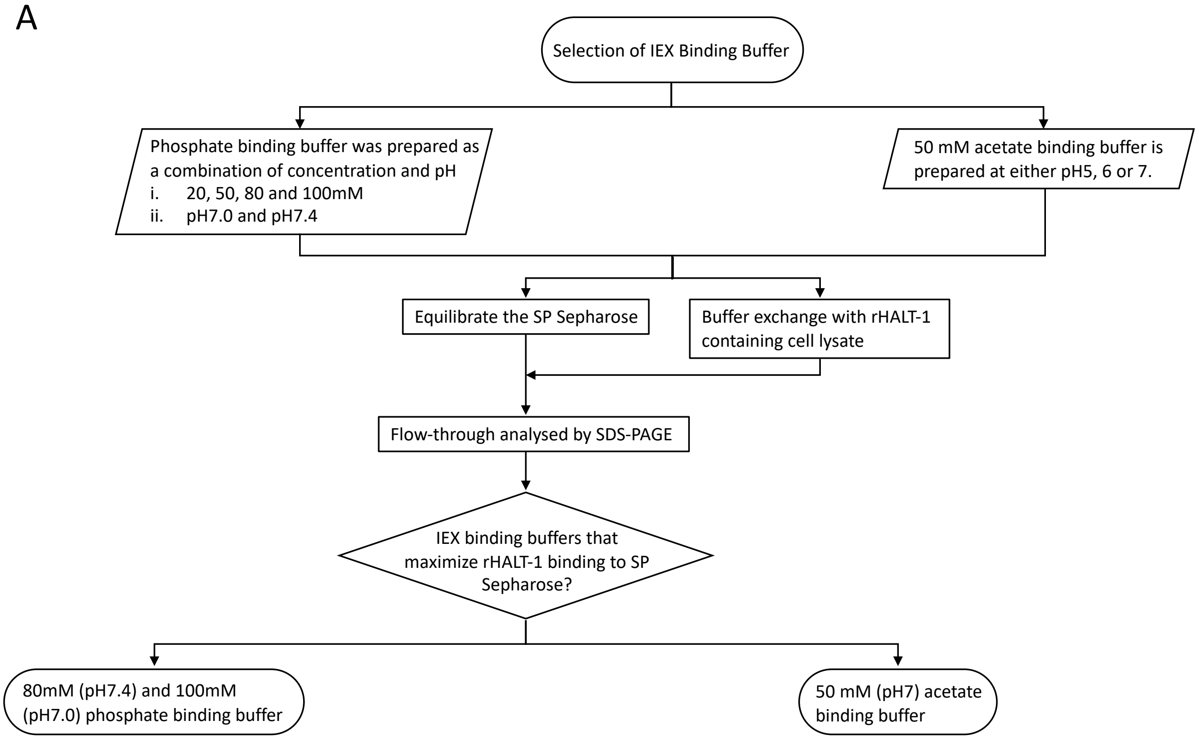

B

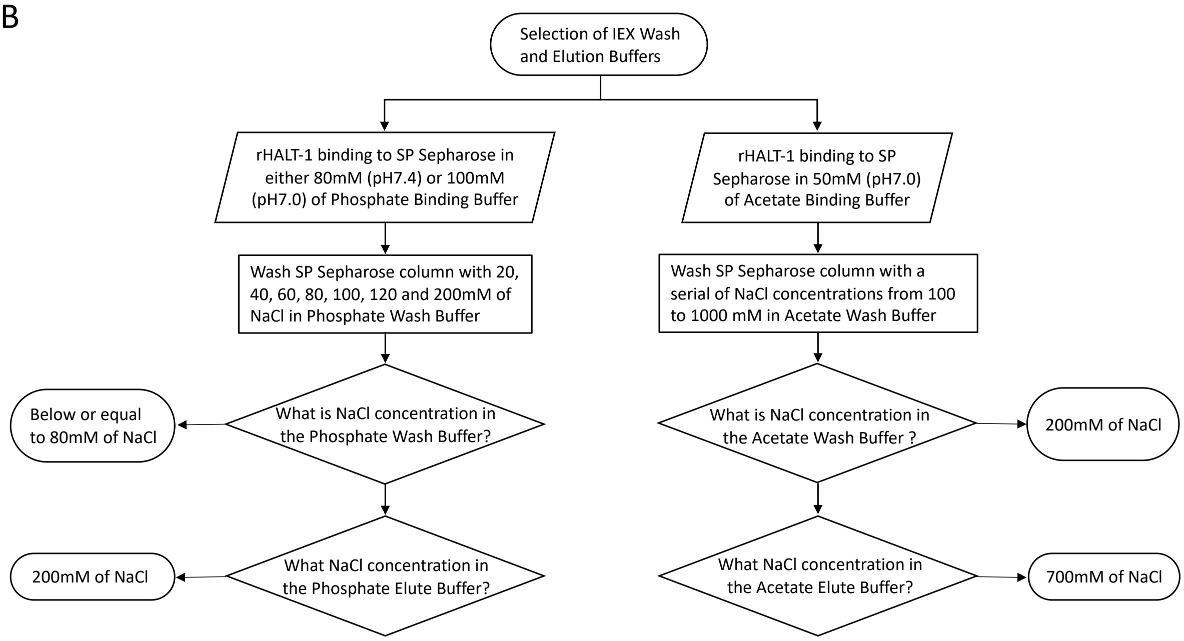

Supplementary Figure S1. Flow chart of the optimization of various buffers used in the ion exchange chromatography of rHALT-1. (A) Optimization of concentration and pH in the Phosphate and Acetate Binding Buffers. (B) Optimization of the NaCl concentration in Phosphate Wash and Elute Buffers and Acetate Wash and Elute Buffers.
