# Supplementary material for: Enhanced production of recombinant HALT-1 pore-forming toxin using two-step chromatographic procedure
Source: MethodsX. 2023 Feb 11;10:102073. doi: 10.1016/j.mex.2023.102073 (PMC9971028; doi:10.1016/j.mex.2023.102073)
Supplement: Supplementary file 2 [file mmc2.docx]

**Supplementary Table S1. Breakdown of the time spent on each step of the two-step rHALT-1 purification**

| **Two-Step Purification** | **Duration (minutes)** | |
| --- | --- | --- |
| **1. Sample Preparation** | | |
| Pulse on | 5 | |
| Pulse off | 15 | |
| Centrifuge (x8,000g sonicated cells) | 10 | |
| Total time | 30 | |
|  | | |
| **2. IMAC (2 mL resin per 50 mL cell culture)** | | |
| Column preparation | 5 | |
| Sample binding | 30 | |
| Washing | 20 | |
| Elution | 4 | |
| Total time | 45 | |
|  | | |
| **3. Buffer exchange** | | |
| Buffer exchange with protein concentrator | 60 | |
|  |  |  |
| **4. IEX (2 mL resin per 50 mL cell culture)** | Acetate buffer | Phosphate buffer |
| Column preparation | 5 | 8 |
| Sample binding | 31 | 20 |
| Washing | 5.5 | 18 |
| Elution | 6 | 2 |
| Total time (IEX) | 47.5 | 48 |
|  | | |
| **5. Buffer exchange** | | |
| Buffer exchange with protein concentrator | 60 | |
|  | | |
| **Grand Total** | **4 h 2.5 m** | **4 h 3 m** |
